# “Let’s Talk Stigma”: A Pharmacy-Based Program for Opioid Use Disorder Anti-Stigma Education in Pennsylvania

**DOI:** 10.3390/pharmacy14010003

**Published:** 2025-12-24

**Authors:** Joni C. Carroll, Sophia M. C. Herbert, Kim C. Coley, Thai Q. Nguyen, Melissa A. Somma McGivney, Kelsey L. Hake, Jennifer Padden Elliott, Elizabeth Bunk Barton

**Affiliations:** 1 Department of Pharmacy and Therapeutics, University of Pittsburgh School of Pharmacy, Pittsburgh, PA 15261, USAcoley@pitt.edu (K.C.C.);; 2 Allegheny County Health Department, Pittsburgh, PA 15219, USA; 3 Center for Integrative Health, Duquesne University School of Pharmacy, Pittsburgh, PA 15282, USA

**Keywords:** stigma, opioid use disorder, pharmacy, mixed methods

## Abstract

Opioid overdoses in the United States remain a significant public health concern. Opioid use disorder (OUD) is stigmatized, exacerbating negative health outcomes. Reducing stigma in healthcare, including in pharmacies, is critical. The “Let’s Talk Stigma” program was collaboratively developed with two schools of pharmacy, a local health department, and individuals with lived drug use experience. It aimed to reduce OUD-related stigma among pharmacists, pharmacy technicians, student pharmacists, and other allied health professionals. The program included six core components: a podcast, continuing education, a standardized curriculum for student pharmacists, training for pharmacy technicians and medical assistants, pharmacy outreach by student pharmacists, and partnerships with chain pharmacies. The anti-stigma podcast reached a global audience with nearly 22,000 listens, while local sessions engaged over 5000 individuals. These initiatives were integrated into Doctor of Pharmacy curricula, with student pharmacists distributing stigma-reduction kits in local pharmacies. A mixed-methods approach, incorporating qualitative data from participant reflections and quantitative data from surveys, podcast analytics, and attendance records, was used for program evaluation. Participants reported increased awareness of stigma, improved attitudes, and greater professional responsibility to reduce stigma. The program successfully leveraged partnerships, flexible delivery methods, and inclusion of people with lived drug use experience in its design.

## 1. Introduction

It is estimated that 73.6 million, or 1 in 4 people aged 12 and older, used illicit substances in the United States in 2024. In 2024, approximately 7.8 million people misused opioids, and 16.8% of Americans were identified as having a substance use disorder in the past year [1]. This continues to be a critical public health concern [2]. These data demonstrate a broader need to support individuals with opioid use disorder (OUD) through harm reduction approaches, including reducing stigma surrounding drug use, as OUD and other substance use disorders are highly stigmatized [3,4]. Harm reduction, as it relates to drug use, is a philosophy of care and public health strategy rooted in social justice principles that aims to reduce drug-related harm (e.g., prevent mortality from accidental overdose, prevent spread of infectious diseases through sterile needle access, decrease stigma, etc.). Stigma negatively impacts health outcomes and leads to lack of retention in patient care, discontinuing treatment, overdoses, and infections [4,5,6,7,8,9,10,11,12,13,14,15]. People who use drugs frequently encounter negative attitudes within healthcare settings, as healthcare providers often express either a lack of readiness or satisfaction when caring for this population [5,16]. Stigma is also found among pharmacy teams, even among those who may provide harm reduction services such as naloxone for reversal of opioid-induced respiratory depression or over-the-counter syringe access [9]. Stigma is a frequent barrier that people who use drugs may encounter in communities [9]. If stigma can be reduced in healthcare settings, individuals who use drugs may be more likely to engage in services for treatment or safer use [10]. Development of educational outreach and training programs to address stigma surrounding substance use disorders among pharmacists and other healthcare team members could improve familiarity with OUD and comfort in engaging with this patient population [4]. These discussions can lead to changes in workplace culture and improve care for individuals with OUD [9]. Reducing experiences of substance use stigma in healthcare settings, including in community health settings like pharmacies, is a key step to improving health outcomes for individuals with OUD [17].

Pharmacists are medication experts who can collaborate with other members of the healthcare team to help patients manage their opioid medication use and treat OUD. Pharmacists are present and accessible in communities across the country, and people visit their pharmacies more frequently than their primary care providers [18]. Frequent interactions with community pharmacy teams can help build patient rapport and trust, which is essential to improving individual and community health. These interactions can allow pharmacy teams to influence patients’ care experiences and address stigma related to OUD, either perpetuating or alleviating it. Pharmacists now offer more OUD-focused services, furthering the need for additional anti-stigma training, such as pharmacy teams may encounter this patient population more often [9,19]. Given their roles as medication experts, their accessibility, and their frequent contact with the community, there is a great need to further educate pharmacists, pharmacy technicians, and student pharmacists to recognize and address OUD-related stigma. 

Reducing OUD-related stigma among pharmacy teams and other allied health professionals is a priority in Allegheny County, Pennsylvania, given the high rates of fatal and non-fatal opioid overdoses. Allegheny County consists of 130 municipalities in the greater Pittsburgh, Pennsylvania area. In 2020, 689 overdoses were reported, which was a 22% increase from 2019 [20]. In 2019, the local health department partnered with the University of Pittsburgh and Duquesne University Schools of Pharmacy to implement strategies to address stigma surrounding OUD among healthcare providers. Together, they designed a program to reduce OUD-related stigma locally and beyond.

### Objective

We aimed to evaluate the reach and impact of an anti-stigma education and dissemination program designed to reduce stigma associated with opioid use disorder among pharmacists, pharmacy technicians, student pharmacists, and allied health professionals. 

## 2. Materials and Methods

### 2.1. Program Design

The University of Pittsburgh and Duquesne University Schools of Pharmacy in Pittsburgh, Pennsylvania, created a program titled “Let’s Talk Stigma” that utilized a mix of educational, dissemination, and pharmacy outreach tactics aimed to reduce stigma among pharmacy teams and other allied health professionals. We define pharmacy teams as pharmacists and student pharmacists and other allied health professionals as pharmacy technicians or those who help perform clinical or administrative tasks in healthcare settings (e.g., medical assistants). This program consisted of six initiatives: (1) “Let’s Talk Stigma” Podcast; (2) Continuing Education for Practicing Pharmacy Teams; (3) Standardized Curriculum for Student Pharmacists; (4) Training Program for Future Pharmacy Technicians and Medical Assistants; (5) Pharmacy Outreach; and (6) Partnership with Chain Pharmacy for Pharmacy Team Education. Program development began in 2019, and all six initiatives were deployed by 2022. Program initiatives were informed by individuals with lived drug use experience who consulted on the initial educational content development. Details of these six initiatives are described below.

#### 2.1.1. “Let’s Talk Stigma” Podcast

We partnered with the Pharmacy Podcast Network to develop and distribute a 7-episode “Let’s Talk Stigma” podcast mini-series designed to address the stigma surrounding OUD [21]. The Pharmacy Podcast Network (PPN) is a podcast platform dedicated to the pharmacy profession, with over 5 million downloads, 4000+ episodes, and more than 30 unique shows since its launch in 2009. The PPN delivers content on clinical care, public health, mental health, business, and pharmacy education. PPN partners with academic institutions and industry leaders to elevate pharmacy voices, reduce stigma, and promote meaningful conversations in healthcare. Its purpose is to serve as an educational, advocacy and professional growth resource for pharmacy. The “Let’s Talk Stigma” podcast included voices of individuals with lived drug use experience and their loved ones, harm reduction experts, and addiction medicine healthcare professionals. Each episode was brief (<24 min each) so busy healthcare professionals could easily listen to the episodes. Episode topics included: (1) the historical context of why stigma exists; (2) treating OUD as a chronic health condition; (3) inter- and intrapersonal stigma; (4) harm reduction and naloxone; (5) medications for opioid use disorder and their associated stigma; (6) the intersection of stimulant use, OUD, and people who experience incarceration; and (7) the intersection of polysubstance use and the unhoused population. The podcast was distributed through Spotify, Apple Podcasts, other podcast RSS feeds, and social media. The project team also disseminated the podcast to student pharmacists at both the University of Pittsburgh and Duquesne University Schools of Pharmacy, through continuing education sessions with pharmacists, to medical assistants and pharmacy technician trainees, and it was promoted by the state pharmacy association on their website and newsletters.

#### 2.1.2. Continuing Education for Practicing Pharmacy Teams

The University of Pittsburgh Duquesne University Schools of Pharmacy disseminated OUD and anti-stigma education to pharmacists through the “Let’s Talk Stigma” podcast, live virtual continuing education programs, webinars with local pharmacy networks, and live professional pharmacy organization events. We partnered with the state pharmacy association to provide pharmacist and pharmacy technician continuing education credits for the previously mentioned 7-episode podcast and virtual educational programming. Live virtual continuing education programs titled “Let’s Talk Stigma: The Opioid Epidemic, Naloxone, and Barriers to Care” reviewed the types of stigma, implicit bias, how stigma can impact individual wellbeing, and how people who use drugs receive healthcare. These sessions included panel discussions with pharmacists from various practice sites. Webinars with a local pharmacy network consisted of training on safe drug disposal and tips for pharmacists on how to reduce stigmatizing language. The webinar recording and additional resources were sent through the pharmacy network newsletters [22]. Live pharmacy organization events included brief discussions on terms to use and avoid when referring to substance use disorder.

#### 2.1.3. Standardized Curriculum for Student Pharmacists 

A standardized curriculum with consistent learning objectives was developed for student pharmacists and deployed at both schools of pharmacy within their Doctor of Pharmacy (PharmD) programs. The PharmD programs prepare students to become licensed pharmacists, and PharmD experiential learning involves hands-on experience in clinical pharmacy settings. The curricular learning activities and didactic lessons were embedded within experiential learning or a professional development PharmD course. Standardized learning activities included: (1) Facilitated discussions about OUD as a chronic health condition; (2) Hearing from individuals with lived experiences who shared their personal stories; (3) Discussion about the importance of using person-first language; (4) Review of local treatment, harm reduction, and recovery resources available; and (5) Participation in online discussion boards about these topics, including self-reflection of key takeaways. Students also completed the Pennsylvania Department of Health and Pennsylvania Department of Drug and Alcohol Programs Online Naloxone Training Self-Study Video [23]. These activities were completed by either second- or third-year student pharmacists at the University of Pittsburgh or Duquesne University, respectively.

#### 2.1.4. Training Program for Future Pharmacy Technicians and Medical Assistants

We also partnered with a career training and academic enrichment center in Pittsburgh, PA, that prepares individuals to become medical assistants and pharmacy technicians. These students were given class time to listen to the “Let’s Talk Stigma” podcast during the first of two sessions. Students shared their written reflections on why stigma exists, their biggest learning points from the podcast, and the roles they may have as future pharmacy technicians and medical assistants in reducing OUD-related stigma. At the second session, trained pharmacist instructors presented additional introductory OUD and stigma content, facilitated an open discussion on the topics, and deployed the state health department’s official naloxone training [23]. Naloxone provided by the local health department was available to hand out to trainees. All lesson plans, presentations, reflection worksheets, podcast MP3s, and handouts were packaged together and provided to program directors for future use, using a train-the-trainer approach.

#### 2.1.5. Pharmacy Outreach by Student Pharmacists 

We assembled “Let’s Talk Stigma Kits” that included print materials about OUD, stigma reduction, and local community resources for harm reduction and treatment. Examples included an overview of local overdose trends and statistics, a Words Matter flyer from the National Institute on Drug Abuse [24], and an Allegheny County Steps of Recovery brochure [25]. Student pharmacists at both schools of pharmacy distributed these kits to pharmacist preceptors at pharmacies in Allegheny County, Pennsylvania. The kits provided pharmacists with materials they could pass along to patients to connect them to existing community resources, and they were also used to spark conversations between student pharmacists and pharmacists about how pharmacy teams can reduce stigma around OUD. Students shared their learnings on OUD and stigma from class (Initiative 3) with their pharmacist preceptors, using the kit materials as a reference for discussion. Additional details of this initiative are published separately [26].

#### 2.1.6. Partnership with Chain Pharmacy for Pharmacy Team Education

The program team partnered with a regional supermarket chain pharmacy to provide educational resources for pharmacy team leaders to conduct anti-stigma training with pharmacy team members. The training highlighted the importance of specific language to use and avoid when talking about addiction and served as an additional venue to distribute the “Let’s Talk Stigma” Podcast. Resources provided to pharmacy teams included a newsletter on the negative impact of stigmatizing language and how to use person-first language to mitigate stigma, as well as resources regarding naloxone and virtual recovery programs that pharmacy teams could refer patients to.

### 2.2. Program Evaluation

We utilized a mixed-methods approach and the CDC’s Framework for Evaluation in Public Health as a guide for programmatic evaluation [27]. Qualitative and quantitative data sources were used to measure program implementation, reach, engagement, and effectiveness (Table 1). Data sources included podcast analytics (listener counts and locations), educational session attendance records, post-educational program questionnaires and written reflections, and the number of pharmacies and individuals reached. Descriptive statistics were used to characterize quantitative data. Qualitative data was available for Initiatives 1–5. An inductive, rapid thematic approach was used to identify themes from qualitative data sources. Three investigators met 10 times to identify themes through consensus discussions. Investigators triangulated participants’ written responses from the qualitative data sources. This project was reviewed by the University’s Institutional Review Board, and it was determined that it did not meet the criteria for human subjects research.

## 3. Results

**Figure 1:**
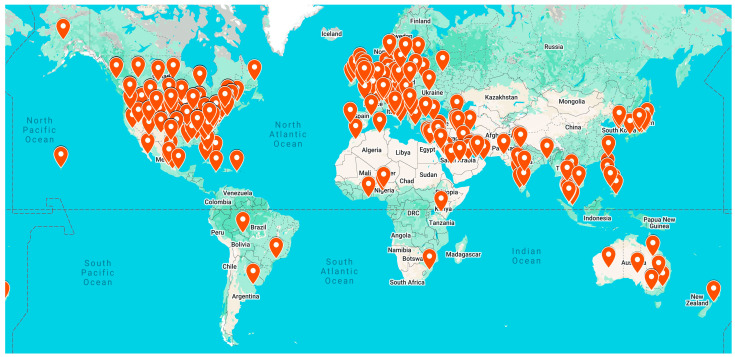
“Let’s Talk Stigma” Podcast Reach—Listener Locations.

All six “Let’s Talk Stigma” initiatives were implemented from 2020 to 2023 according to original plans, with some shifts in the timeline and delivery mechanisms due to the COVID-19 pandemic (e.g., some programs were deployed virtually instead of in-person). This program reached over 5000 individuals locally in four years (Table 2). Additionally, the Let’s Talk Sigma Podcast alone had nearly 22,000 listens as of August 2023. The podcast reached people from 66 countries and had listeners in all 50 U.S. states and the District of Columbia (Figure 1). This podcast is still broadly available to the public. An evaluation of the podcast effectiveness [21] (Initiative 1) and additional details about the Pharmacy Outreach by Student Pharmacists (Initiative 5) are reported elsewhere [26].

Program participants reported an increased awareness of stigma, the need for compassion and empathy, changes in attitudes, and a professional responsibility to reduce stigma. Results of our thematic analysis are found in Table 3. Additionally, participants reported they enjoyed the format of the podcast, live discussions, and content within each initiative. For example, one pharmacist stated they “really enjoyed the format of this continuing education—was able to listen to podcasts during my commute and gain a valuable perspective on this topic.” A medical assistant trainee also stated, “I wish this [training] was more public and everyone should take it.” Both the University of Pittsburgh and Duquesne University Schools of Pharmacy continue to incorporate “Let’s Talk Stigma” programming into their PharmD curricula for student pharmacists.

## 4. Discussion

### 4.1. Lessons Learned

We developed and deployed a comprehensive “Let’s Talk Stigma” anti-stigma program for pharmacists, student pharmacists, and allied health professional trainees with the goal of addressing the stigma surrounding OUD in Allegheny County, PA. Our program was novel because it was designed to reach both practicing clinicians and learners still in training with anti-stigma education. The evaluation of this program demonstrates the program’s substantial reach both locally in Pennsylvania and globally through the podcast dissemination, engagement of partners and participants, and the positive impact on learners. We believe this program was successful because of the extensive collaboration between multiple partners and the involvement of individuals with lived drug use experience. There were several key lessons learned over the four years of program implementation. It is common to present “lessons learned” as part of traditional public health program evaluation, so others can replicate key aspects of our program. These lessons included involving people with lived drug use experience in the design of the program, partnering with various organizations to amplify program reach, and utilizing the existing infrastructure of Schools of Pharmacy experiential learning programs to engage pharmacists in the program. 

Most importantly, we involved individuals with lived drug use experience from the beginning of the program design to ensure our content and materials were relevant and culturally appropriate. Involving people with drug use experience in the development or delivery of healthcare services has the potential to reduce stigma [28]. Storytelling by individuals with lived drug use experience within the podcast was noted by program participants as being an effective strategy to bring experiences of stigma “to life.” Storytelling as an approach to reducing stigma is supported within the literature [29,30], though it may not be as effective in every setting [31]. The use of a podcast emerged as an innovative mechanism to circulate anti-stigma messages to healthcare personnel and could be used with multiple audiences and levels of learners. Draft audio files of the podcast were shared with interviewees to ensure the content met key learning objectives. We feel this was a critical step to ensure the educational messaging was accurate and appropriate for the audience. Including stories from individuals with lived drug use experience was also emphasized by student learners as meaningful to their learning and was highlighted in our program evaluation data. The use of podcasts for training health sciences professionals and learners continues to expand and may be an efficient way to expose various learners to individuals with lived/living drug use experience [32,33].

Second, partnerships played a crucial role in the program’s success. Collaboration between a health department and two Schools of Pharmacy amplified this program’s reach and impact, as evidenced by the large number of individuals engaged across the six program initiatives. Academic health departments, which represent mutually beneficial collaborative working relationships between health departments and academia, continue to grow [34]. These relationships enhance student learning in public health, reinforce implementation of evidence-based public health practices, and address real-world issues [35]. Schools/colleges of pharmacy in particular can offer numerous resources to support health departments with implementation of public health programs like “Let’s Talk Stigma.” The Schools of Pharmacy engaged in this program had pre-existing practice relationships with clinically integrated networks of community pharmacies, hundreds of experiential learning sites and preceptors, the Pharmacy Podcast Network, the state pharmacy association, and health plans. Schools/colleges of pharmacy can also engage personnel like student pharmacists, faculty, staff, other health professionals, and post-graduate learners for program dissemination and outreach efforts. Globally, most pharmacy-based education includes some experiential or “on-the-job” training experiences, which can be leveraged when developing anti-stigma training programs for OUD or other stigmatized disease states.

Connecting with pharmacists in practice through experiential learning programs (e.g., student clinical rotations at pharmacies) can help distribute public health information into communities. The “Let’s Talk Stigma” program initiatives served as both educational activities for learners and rapid dissemination strategies for our local public health department. We integrated educational programming alongside student outreach into real-world pharmacy settings. This approach utilized established partner connections, avoiding the need to recreate public health systems of outreach from scratch. Students can gain knowledge from their participation in these hands-on learning experiences as well. An additional benefit of working with health professions learners is reaching them with anti-stigma education before they enter practice [36]. This is critical so they may be informed before becoming influenced by existing attitudes and stigma among people who did not receive this education within their formal training programs [5,8,36].

There are several limitations to our program design and evaluation. Most of our programming occurred during the COVID-19 pandemic, when pharmacy teams were busy providing vaccinations to the public. This may have limited attendance at continuing education sessions and influenced the number of responses on post-education questionnaires from pharmacists. The medical assistant and pharmacy technician student classes were blended together, and some pre-and post-program questionnaires did not ask the students to differentiate their discipline, which limited our ability to compare differences between students in the two fields. Additionally, it is difficult to measure stigma reduction; however, we feel our participant quotations and engagement with program initiatives demonstrate movement towards decreasing stigma. There may be new tools to measure stigma among pharmacy teams [37]. Despite the “Let’s Talk Stigma” program’s success, it is important to recognize that stigma surrounding OUD persists and will require sustained anti-stigma educational efforts over time [36].

### 4.2. Implications for Policy and Practice

- Incorporating individuals with lived drug use experience into the initial design of anti-stigma educational programming is important to ensure program content is culturally appropriate.
- Partnerships between Schools/Colleges of Pharmacy and local health departments can maximize the reach and impact of anti-stigma educational programs for pharmacy teams and allied health professionals.
- Anti-stigma programs around OUD and drug use should continue to be developed and funded on an ongoing basis in the United States. These programs should include pharmacists and other health professionals in their development and deployment.

## 5. Conclusions

While our program was successful, anti-stigma educational efforts directed towards healthcare professionals need to continue to be prioritized and funded. It is imperative that schools/colleges of health sciences embed anti-stigma education within curricula. Additionally, we recommend that state and local health departments continue to partner with schools/colleges of health sciences to further publicize anti-stigma education.

## Figures and Tables

**Table 1:** Program Evaluation Questions, Indicators, and Measures.

Evaluation Questions	Indicators and Measures
**Implementation**—Were the six initiatives implemented as planned?	Completion status of each initiative. Adherence to the timeline for deployment. Changes to implementation during rollout.
**Reach and engagement**—How many individuals were reached, and were they engaged in each initiative?	The number of podcast listens per episode. Attendance numbers for CE programs, webinars, and live events. Participation rates in training sessions for pharmacy technicians and medical assistants. Distribution and utilization of stigma reduction kits by student pharmacists. Number of pharmacy team members reached in the chain pharmacy partnership.
**Effectiveness**—What did participants learn? Did the initiatives lead to changes in attitudes among participants or a reduction in stigma among participants?	Pre- and post-program questionnaires. Evaluation of knowledge gained (e.g., through worksheets or quizzes). Post-educational program written reflections.
**Sustainability**—What mechanisms are in place to sustain the initiatives beyond the program’s end?	Ability to use resources developed beyond the initial project period (e.g., podcast episodes, training materials). Integration of stigma reduction content into ongoing training or curricula.
**Improvement**—What improvements can be made to increase the program’s impact?	Feedback from participants on program components. Analysis of gaps or challenges faced during implementation.

**Table 2:** “Let’s Talk Stigma” Program initiatives and their reach as of August 2023.

Program Initiative	Program Initiative Reach
1. “Let’s Talk Stigma” Podcast	21,928 listens
2. Continuing Education for Practicing Pharmacy Teams	791 pharmacists or pharmacy technicians
3. Standardized PharmD Curriculum for Students Pharmacists	1010 student pharmacists
4. Training Program for Pharmacy Technicians and Medical Assistants	15 learners
5. Pharmacy Outreach by Student Pharmacists	484 pharmacists, pharmacy support staff, or other employees 197 pharmacies *
6. Partnership with Chain Pharmacy for Pharmacy Team Education	780 pharmacists 1997 pharmacy support staff (technicians, interns, cashiers), and 214 pharmacies *

* Some pharmacies may have overlapped between groups; in such cases, they were exposed to both initiatives.

**Table 3:** Qualitative Program Evaluation Results.

Themes	Participant Quotes
**Awareness of the Impact of Stigma**: Stigma surrounding OUD is pervasive, even within healthcare. It is important for healthcare practitioners to recognize and take steps to mitigate stigma.	“I think [stigma] creates barriers for people to seek treatment and access to care.”—allied health profession student * “A compelling topic of discussion was the origin of stigma surrounding drug users… I think understanding where stigma comes from helps to undo it, if we can identify why we have an intrinsic bias or a stigmatized view of a person we are better able to reverse that way of thinking.”—student pharmacist “The stigma within all healthcare disciplines is widespread. Pharmacists and pharmacy technicians are part of a crucial access network for patients to receive appropriate medication counseling, naloxone, and valid opioid prescriptions. Patients may not feel comfortable if healthcare workers demonstrate stigma in their body language or words.”—student pharmacist
**Empathy and Compassion**: Empathizing with people who use drugs may lead to better, more compassionate care provided by healthcare teams.	“I think that it’s our job to be that kind and understanding person that the people who struggle with drugs can confide in when they’re ready for help. We need education and empathy to make their experience safer and positive.” —allied health profession student * “The presentation was good at helping one to see the impact on the patient and very good at eliciting empathy.”—pharmacist “... Just realizing that you never know what someone is going through, and one act of kindness can completely change that person’s attitude or decisions.”—student pharmacist
**Professional responsibility**: Pharmacists and other healthcare practitioners can play a key role in treating OUD and addressing the stigma attached to it.	“Pharmacy techs and medical assistants have a huge role to play in reducing stigma because they are the first people to talk to them. I would not judge them and treat them as a human. I would help them in any way I could.—allied health profession student * “If coworkers are talking disrespectfully about people with substance use disorders, it is up to us to call that out or defend the person.”—allied health profession student * “Community pharmacists play an important role in assisting patients with substance use disorders, through dispensing necessary medications and counseling on how to use these medications properly. In order to provide the best care possible, it’s very important that all healthcare providers use respectful language and avoid stigmatizing languages when speaking with patients.”—student pharmacist
**Changing attitudes:** Anti-stigma training can lead to positive shifts in attitudes of healthcare providers and can help them view patients with OUD as individuals facing a health challenge.	“I have a clearer picture of what it is, and I was struck by how similar diabetes and opioid use disorder is. When you think of it that way, it makes perfect sense. It is the analogy I will use to speak to my family about destigmatizing drug use.”—MA 2 “Informative, I learned new terms I plan to incorporate.”—pharmacist “I always thought of myself as an empathetic person but realized that I might have had a stigma too. I need to start thinking about this disease differently and do better.”—student pharmacist “...As someone who does not personally know someone affected by opioid use disorder, I feel more prepared and slightly less intimidated on how I would handle a situation working to help patients with [opioid use disorder].”—student pharmacist
**Importance of personal stories:** Anti-stigma training that includes personal stories from individuals with lived drug use experience engages people in the training content and fosters empathy towards people with OUD.	“Hearing [the person’s] stories that she experienced was an eye-opener, especially if you don’t know anyone personally who struggles [with addiction]”—allied health profession student * “Well done. I enjoyed the stories. It has made me think differently about dispensing suboxone, for the better.”—pharmacist “... Listening to stories of negative encounters with healthcare providers as people are going through recovery had an impact on me and inspired me to be a better example of our profession and do better for this patient population.”—student pharmacist “My favorite quote from the podcast was “no one wakes up in the morning and chooses to battle addiction.” Hearing from real people with substance use disorders in the podcast helped me realize (more so than I had before) that this disorder is a disease and should be treated as such.”—student pharmacist
**Harm reduction matters**: Harm reduction strategies can help people with OUD and can take various forms. These include choosing a less stigmatizing language when discussing OUD and offering resources such as naloxone, medications for treatment, and access to syringe services.	“Don’t limit access—come up with a solution. Make naloxone more available (access and cost), educated and be more public, offer options, syringe service programs…I never heard of some of these services before this podcast, which is unfortunate because who else doesn’t know these exist?”—MA or Pharmacy Technician “I learned that the way you discuss naloxone can impact the patient’s willingness to receive it.”—student pharmacist “I have learned that opioid use disorder is a chronic disease that is complex. As pharmacists, we need to shift focus from controlling access to medication to risk reduction strategies to help these individuals. There are many factors in a patient’s life that lead to substance use disorder such as stress or trauma. We need to consider all those factors and help patients…”—student pharmacist “One takeaway I had from the podcasts was the significant role pharmacists can play in selling syringes. Many pharmacists raise prices on syringes or only sell the syringes in boxes of large quantities. This is part of stigmatizing those who suffer from addiction and can make them more prone to diseases like hepatitis and more which can be detrimental to their health.”—student pharmacist

* Allied health profession student = medical assistant or pharmacy technician student.

## Data Availability

The data presented in this study are available on request from the corresponding author due to the qualitative data restrictions in accordance with the informed consent provided by participants and the terms of ethical approval.

## Abbreviations

The following abbreviations are used in this manuscript:

OUD	Opioid Use Disorder
PPN	Pharmacy Podcast Network
PharmD	Doctor of Pharmacy
